# A Sub‐Microsecond Switch Enabling SWIFT ^23^Na Imaging at 10.5 T

**DOI:** 10.1002/mrm.70461

**Published:** 2026-06-08

**Authors:** Russell L. Lagore, Simon Schmidt, Edward J. Auerbach, Naoharu Kobayashi, Christoph Schildknecht, Steen Moeller, Elnaz Mahmoudi Mahmoudalilou, Štefan Zbýň, Jutta Ellermann, Gregor Adriany, Gregory J. Metzger

**Affiliations:** ^1^ Center for Magnetic Resonance Research (CMRR) University of Minnesota Minneapolis Minnesota USA; ^2^ German Cancer Research Center (DKFZ) Heidelberg Germany; ^3^ ETH Zürich, Institute for Biomedical Engineering ETH Zurich and University of Zurich Zurich Switzerland; ^4^ Department of Biomedical Engineering, Cleveland Clinic Research Cleveland Clinic Cleveland Ohio USA

**Keywords:** cartilage, sodium MRI, UHF, ultra‐high field MRI, X‐nuclei

## Abstract

**Purpose:**

To enable sodium SWIFT imaging, which is a zero‐echo time imaging technique, at ultra‐high magnetic fields through the development of custom electronics hardware, and to showcase this capability with in vivo imaging results.

**Methods:**

The custom hardware developed consists of a high‐speed optical trigger with 10 ns resolution, an in‐bore PIN diode driver capable of sourcing high current, and RF switches (both an in‐line switchable attenuator and transmit/receive switch) optimized to achieve sub‐microsecond switching speeds while also producing isolation between the transmitter and receiver.

**Results:**

Practical switching speeds of 0.6 μs (receive to transmit) and 1.7 μs (transmit to receive) are achieved on the RF switches, limited by transient‐induced spurious emissions from the low noise amplifier. Transmit to receive isolation of 115 dB is achieved over a 1 MHz bandwidth, with 120 dB of isolation at the Larmor frequency. This was crucial to suppress unblanked RFPA noise. SWIFT images of the human wrist with 1.5 mm isotropic resolution were acquired in less than 5 min to demonstrate the utility of sodium SWIFT imaging at ultra‐high field. SWIFT imaging signal‐to‐noise ratio in a reference phantom with heterogeneous sodium concentrations was comparable to ultra‐short echo time imaging, which provided assurance that hardware had achieved the necessary specifications.

**Conclusions:**

The feasibility of sodium SWIFT imaging at ultra‐high field was established, and a musculoskeletal imaging application was demonstrated. The success of this work enables further development of SWIFT at high and ultra‐high fields for imaging of sodium, proton, and other nuclei.

## 1 Introduction

Magnetic resonance imaging and spectroscopy are valuable molecular imaging modalities providing unique information, but are restricted by the inherently low SNR. That being said, the breadth of both endogenous and exogenous molecular imaging contrasts, in combination with high‐resolution and contrast anatomic imaging, motivates the continued development of the methods and their biomedical application and clinical translation. The promise to improve MRI sensitivity to markers of molecular dynamics, metabolic functional parameters, energetics, and cellular homeostasis, among others, continues to drive the development of molecular imaging methods to higher and higher magnetic fields.

It has been shown that sensitivity, as measured by the signal‐to‐noise ratio (SNR), scales supralinearly for ^1^H MRI with the static field strength (i.e., SNR ∼ B_0_^1.65^) [1], which can be used to increase spatial and/or temporal resolution [2]. While acquisition and anatomy‐specific relaxation effects need to be accounted for, this underlying increase in SNR plays an important role in the overall benefits of ultra‐high field MRI. In the case of protons, transverse relaxation times decrease and longitudinal relaxation times increase in the frequency range of interest, which can work against the SNR advantages. In the case of nuclei other than protons (i.e., x‐nuclei), the natural abundance and overall sensitivity are much lower; therefore, methods to maximize the achievable SNR are of paramount importance. In many cases for x‐nuclei, the impact of relaxation is either neutral [3] (i.e.,^17^O) or advantageous [4, 5] (i.e., ^23^Na), allowing the full benefit of increased SNR at higher field strengths to be realized.

Overcoming the low sensitivity of low‐gamma nuclei such as sodium (^23^Na) is necessary to increase the resolution and decrease the acquisition times for practical integration into a multiparametric, multinuclear exam. With the advent of higher field strengths and improved methodology, the benefit of sodium MRI is being explored in the study of stroke [6, 7] and tumor detection [8], for breast cancer diagnosis [9, 10] and therapeutic response [11], and for the assessment of osteoarthritis [12, 13, 14, 15, 16], muscle [17], kidney function [4, 18, 19], and multiple sclerosis [20, 21, 22, 23]. Sodium's nucleus has a spin 3/2 and experiences a biexponential *T*_2_ relaxation in tissue due to its quadrupolar moment, with both a short and long component of $T_{2}^{*}$ ($T_{2S}^{*}$ and $T_{2L}^{*}$, respectively). The $T_{2S}^{*}$ of sodium can be on the order of only a few milliseconds in muscle tissue at 3 tesla [24] and musculoskeletal (MSK) imaging of sodium at 10.5 T has estimated that $T_{2S}^{*}$ can be as low as 0.4 ms in cartilage of the pediatric knee [25, 26]. At these extremely short relaxation times, the signal decays significantly before it can be acquired using standard imaging sequences. Consequently, previous sodium imaging work has employed ultra‐short echo time (UTE) [27] and zero echo time (ZTE) [28, 29, 30] imaging techniques. For imaging that involves quantifying tissue sodium concentrations, even ultra‐short echo time sequences can result in large biases in these measurements, which may be reduced with ZTE sequences [31, 32].

SWIFT, or Sweep Imaging with Fourier Transform, is a zero echo time MR imaging technique introduced in 2006 [33] and has seen extensive use at 4 T for human imaging [34, 35, 36] and 9.4 T for pre‐clinical imaging [37, 38, 39, 40, 41, 42]. It has demonstrated the ability to image tissues with rapidly decaying signals, such as bony structures and teeth [33, 43, 44]. Consequently, SWIFT has been proposed as an alternative to UTE and ZTE techniques for sodium imaging [25, 45]. Dissemination and use of SWIFT acquisitions, however, have been limited by the specifications of the hardware, which are typically not supported by standard clinical and pre‐clinical system hardware [46].

**FIGURE 1:**
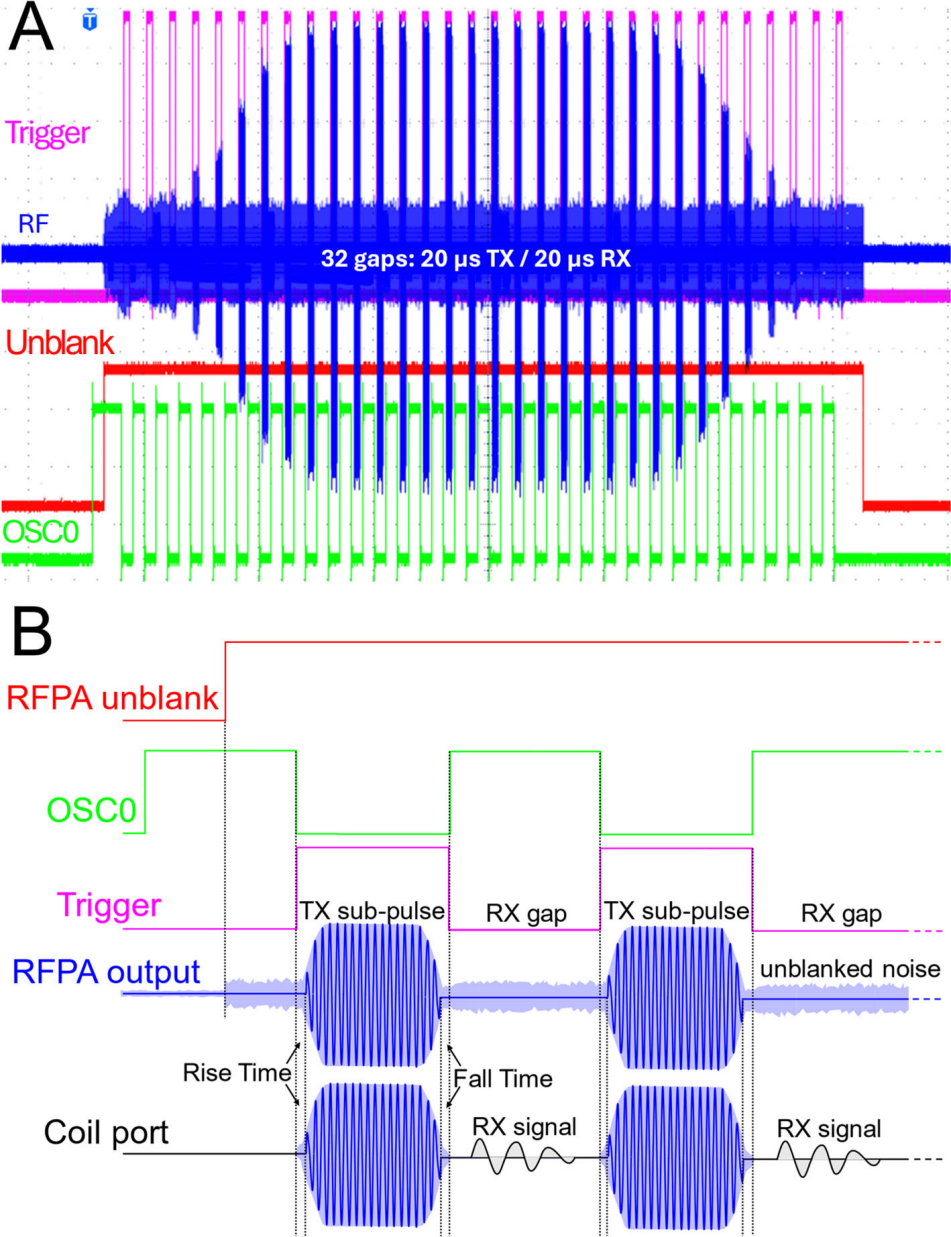
(A) Oscilloscope trace showing a gapped RF pulse as measured at the RFPA output (RF, in blue). A large increase in noise occurs when thed RFPA unblank (red) goes high for the duration of the pulse. Transmit sub‐pulses occur when the trigger (magenta) is high, and the amplitude follows the envelope of the transmit pulse, while receive gaps are interleaved with the transmit sub‐pulses, while trigger is low. The envelope of this RF pulse is a flattened hyperbolic secant (HS4) pulse. Also shown is OSC0, which is the system‐supplied TTL signal. (B) Two transmit/receive gap periods with colors consistent with (A). Depicted are the finite switching times from receive to transmit (rise time) and transmit to receive (fall time), as well as the RF signals at the coil port. RFPA noise is attenuated at the coil port so that the RX signal can be detected in receive gaps.

A gapped transmit pulse, as demonstrated in Figure 1A (blue trace), is employed for the SWIFT imaging [47] used in this work, though other variations of the sequence exist, such as multiband SWIFT [48] and continuous SWIFT (which is technically extremely challenging) [49]. During the gapped transmit pulse, the NMR signal is sampled for brief periods of time between the transmit pulses (illustrated in Figure 1B). Consequently, the MR system is rapidly switched between transmit and receive modes. The sequence described herein demands that the system switch 64 times to accommodate 32 transmit sub‐pulses and receive gaps, each of which is on the order of 20 μs (though this duration can be adjusted). This necessitates rapid switching on the order of one microsecond to minimize dead time. The Siemens MAGNETOM 10.5 T system used in this work, and all Siemens clinical systems, cannot accommodate this rapid switching. While there has been previous hardware development for ZTE imaging at 7 T [50], each vendor's hardware has its own specifications and limitations. Previous attempts at implementing SWIFT at 7 T on the Siemens MAGNETOM system architecture were described [46, 51], but the hardware developed had reliability issues and power handling limitations that prevented full adoption. The hardware necessary to enable SWIFT includes PIN diode drivers which can source high current to enable fast T/R switching, a T/R switch optimized to exploit a high current supply driver with a PIN diode of the correct carrier lifetime and intrinsic region width, and a high‐resolution trigger signal that allows fine control of the sub‐pulse timing for the SWIFT gapped transmit pulse (illustrated by the magenta trace of the trigger signal in Figure 1). Finally, because the RF power amplifier (RFPA) in typical human MR scanners cannot be rapidly blanked during receive, this hardware must also provide high isolation between transmit and receive to ensure that noise from the unblanked RFPA is not injected into the received signal. This high level of noise from the RFPA is also illustrated in Figure 1. All these technical hurdles are cleared in the work presented.

## 2 Methods

**FIGURE 2:**
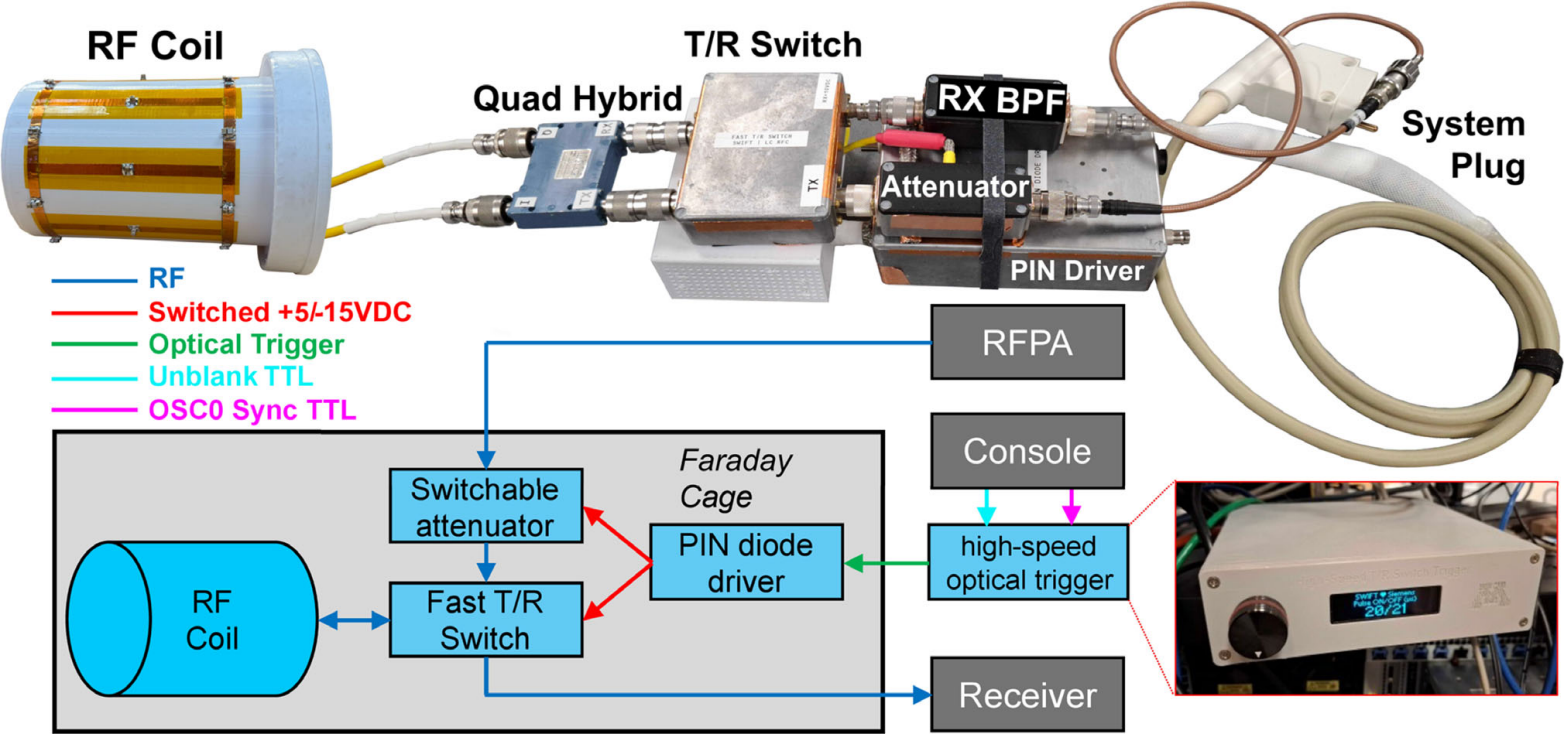
Block diagram of the ultra‐fast switching hardware. Custom hardware developed in‐house is shown in blue. Items in gray are OEM components of the Siemens console, while the RF coil, while being custom‐built in‐house, is not specially developed for SWIFT applications.

Hardware capable of SWIFT imaging on our Siemens 10.5 T system was developed without the need to exchange major system components such as the RFPA—thus maintaining the ability to use existing patient safety controls. The additional hardware developed includes a high‐speed optical trigger, an in‐bore PIN diode driver, a switchable RFPA attenuator, and a fast T/R switch. As all components are designed as extensions to the existing RF transmit chain, SWIFT can be supported without the need for recalibration of the RF Amplifier. Various phantoms and coils were also developed. A photo of the hardware and a block diagram of the system are provided in Figure 2. An earlier prototype of this hardware, developed in 2019, was previously described for 7 T [51], while the in‐bore PIN diode driver was developed and presented in 2023 [52].

### 2.1 High‐Speed Optical Trigger

The ultra‐fast optical trigger was developed in‐house. It accepts the OSC0 TTL signal at the onset of the RF pulse from the Siemens console. The TTL is used as an input from which it directly generates secondary pulses to synchronize the RF hardware's transmit and receive modes as necessary with the intended gapped RF pulse. These pulses are directly generated from OSC0 using a series of monostable multivibrators (or “one shots”), which use advanced high‐speed CMOS (74AHCT series) digital logic gates. The optical output employs a 650 nm transmitter and receiver (HFBR‐x528, Broadcom, Palo Alto, CA, USA). Pulse timing is communicated to the high‐speed optical trigger box via the network from the console. An ATmega microcontroller is used for this communication. The microcontroller also sets timing by adjusting digital potentiometers over the I2C bus. This sets the delay and pulse length timing. After these potentiometers are set, the system is stable, and no further involvement of the microcontroller is necessary; no clock synchronization is required. The timing can be adjusted with a resolution of 10 ns and is manually calibrated for a range of gap lengths. The output from the trigger box is necessarily optical since TTL signals are prone to reflections with resultant distortion to the rising and falling edges of the trigger.

### 2.2 PIN Diode Driver

**FIGURE 3:**
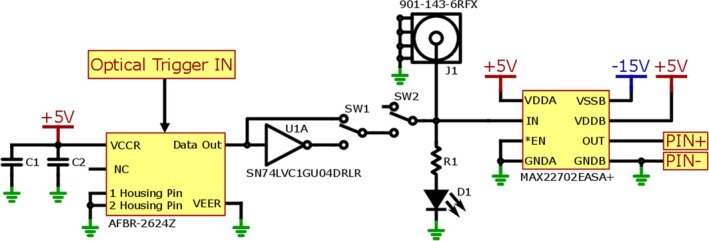
Simplified schematic of the in‐bore PIN diode driver necessary for the fast‐switching RF hardware.

Optical pulses from the high‐speed trigger are used as an input to the PIN diode driver, which is situated within the bore of the 10.5 T magnet. The PIN diode driver requires +7 VDC and −18 VDC supplied via a filter panel from a power supply outside the magnet room. These are, in turn, regulated down to +5 VDC (LD29300P2MTR, STMicroelectronics, Geneva, Switzerland) and −15 VDC (LT3015IMSE, Analog Devices, Wilmington, MA, USA), respectively. The driver used is the Analog Devices MAX22702EASA+. In transmit mode, the driver supplies a constant +5 V and can source over 2 A to forward‐bias the PIN diodes of the attenuator and T/R switches. In receive mode, the driver produces a constant −15 V to reverse‐bias PIN diodes. Eight independent driver channels are furnished to allow for multiple RF channels to be switched. Everything is housed inside a large 188 × 120 × 56 mm^3^ diecast aluminum enclosure (1590D, Hammond Manufacturing Ltd., Guelph, ON, Canada). A simplified schematic is presented in Figure 3.

### 2.3 RF Switch

RF switching between transmit and receive is accomplished with two distinct devices that are designed along a common design philosophy. The first device is a switchable RFPA attenuator, which is placed between the RFPA and the transmit port of the T/R switch. The second device is the T/R switch, with its integrated preamp, which is what fundamentally switches the coil between the transmitter (RFPA) and receiver. The MA4P504‐1072 T (MACOM, Lowell, MA, USA) PIN diode was selected for both RF switches on the basis of PIN diode testing conducted previously [51]. This testing is also described in Supporting Information.

### 2.4 Switchable RFPA Attenuator

The RFPA is unblanked for the duration of the gapped transmit pulse, even during the gaps when the NMR signal is being sampled in receive mode. The consequence of this is that the NMR signal can be easily overwhelmed by the noise of the RFPA. To ensure this does not happen, a fast‐switching attenuator was developed. This two‐port device is placed in‐line between the transmitter and the T/R switch to supplement the T/R isolation of the T/R switch. The principle of operation is simple: during transmit mode, the attenuator should produce minimal insertion loss to the transmit pulse, while in receive mode, the attenuator should produce high attenuation between the input and output ports.

**FIGURE 4:**
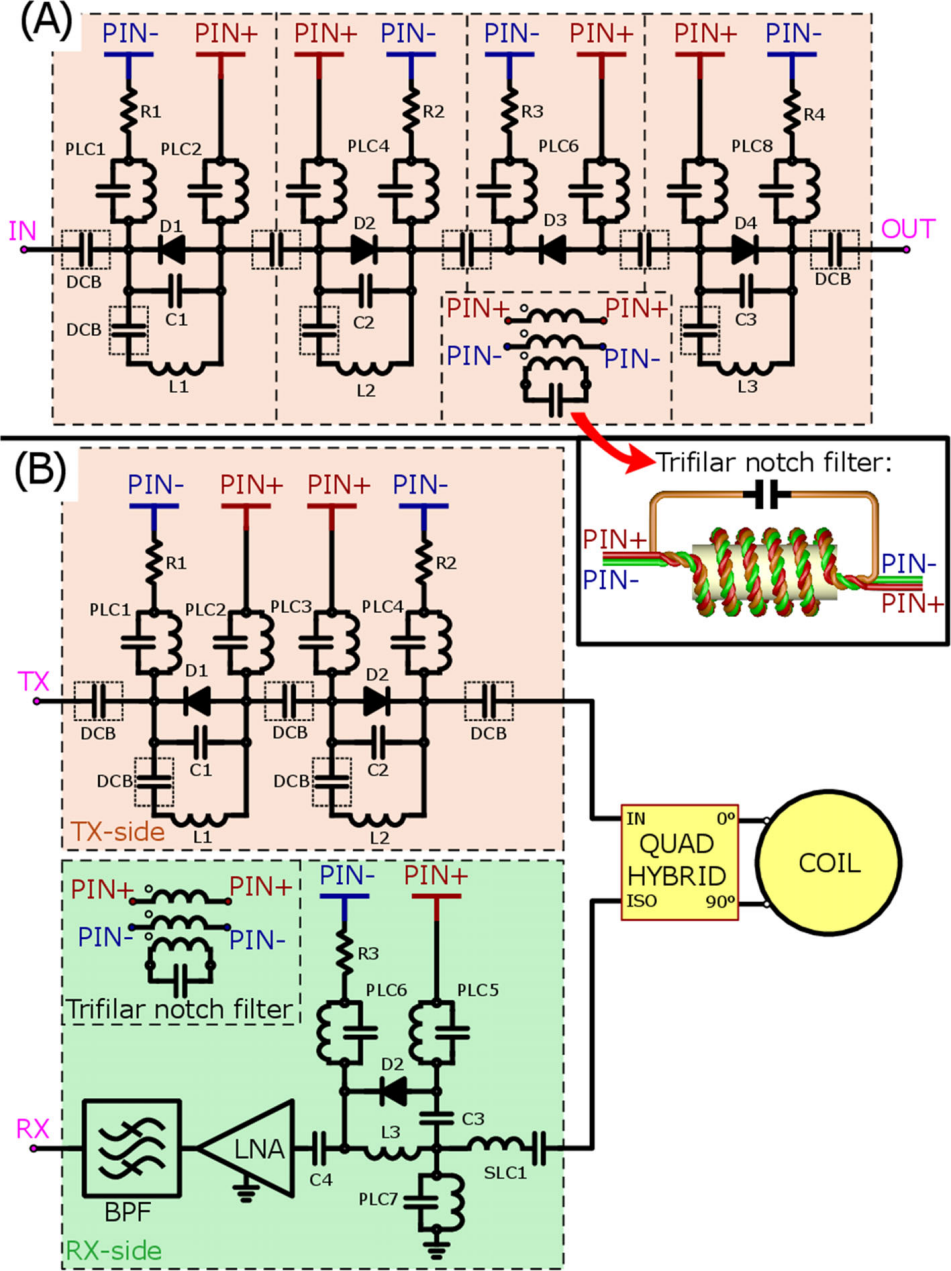
Schematics of (A) switchable attenuator and (B) T/R switch. The inset in (B) is a diagram of the trifilar wound notch filter used for PIN diode bias filtering at RF shield box penetrations to the attenuator and T/R switch. The red and green wires represent the PIN+ and PIN‐ signals, with the orange wire being tuned to the Larmor frequency with a capacitor to produce a high impedance at this frequency. The three inductors made up by the trifilar winding have a high coupling coefficient, so this high impedance is presented on the red and green windings and acts like a band stop or notch filter with high in‐band attenuation.

The attenuator (schematic shown in Figure 4A) is constructed inside a small 93 × 39 × 31 mm^3^ extruded aluminum box (1590ABK, Hammond Mfg). The attenuator consists of four PIN diodes, which are supplied with a switching current via a network of LC resonant RF chokes and bypass capacitors. Each diode receives its bias current in parallel with the others. A 36 Ω resistor is used to limit the current draw of each diode to ˜100 mA. Two PIN diodes face each other in opposite directions and sit in series on the attenuator. When reverse‐biased, they present a near open circuit except for some small capacitive reactance presented by the diodes. For two of four diodes, the capacitance is resonated out using an inductor to achieve a very high real impedance in‐band.

### 2.5 T/R Switch

The T/R switch (schematic also shown in Figure 4B) interfaces the RF coil to the MRI system, providing an integrated low noise amplifier (LNA) on receive and protection of the LNA during transmit. It is also housed inside a 120 × 94 × 34 mm^3^ extruded aluminum box (1590BB, Hammond Mfg). Switching is achieved with three PIN diodes, two in series but opposing directions on the transmit side and one on the receive side, which controls an LC tank circuit in front of the LNA. Diodes are biased in the same way as in the attenuator, using a network of LC resonant RF chokes, bypass capacitors, and current‐limiting resistors, with all diodes driven in parallel to optimize switching speeds. The transmit side of the switch, consisting of a pair of PIN diodes run back‐to‐back, produces an open circuit during receive through back biasing of the PIN diodes while producing a through connection to the coil via forward biasing in transmit mode. Because of the relatively high series capacitance of this PIN diode, excessive noise from the RFPA can leak through the back‐biased diodes of the T/R switch even in receive mode. As such, one of the two diodes on the transmit side of the switch contains an LC resonant tank across the junction. This resonant tank produces additional narrowband T/R isolation at the Larmor frequency.

The integrated LNA is the (now obsolete) SPF5122Z (Qorvo, Greensboro, NC, USA), which achieves 25 dB gain at 118 MHz. An RF clamping diode (UMX9989, Microchip, Chandler, AZ, USA) is placed at the preamp input to ensure it is protected from transmit power. A Chebyshev band‐pass filter is implemented on the LNA output, where the insertion loss has minimal impact on the system noise figure. This bandpass filter was primarily used to clean up the low‐frequency switching transients and pass only the desired in‐band NMR signal.

### 2.6 RF Filtering of the PIN Diode Bias

The PIN diode driver bias signal is provided to the PIN diodes of the RF switching hardware via a shielded twisted pair. A trifilar wound LC notch filter (the drawing of which is shown in the inset in Figure 4B) is implemented at the penetration to each shielded box. This filter rejects frequencies around the Larmor frequency of 118.25 MHz, reducing crosstalk between the RF hardware and the RF coil, thus preserving T/R isolation.

### 2.7 RF Coil

**FIGURE 5:**
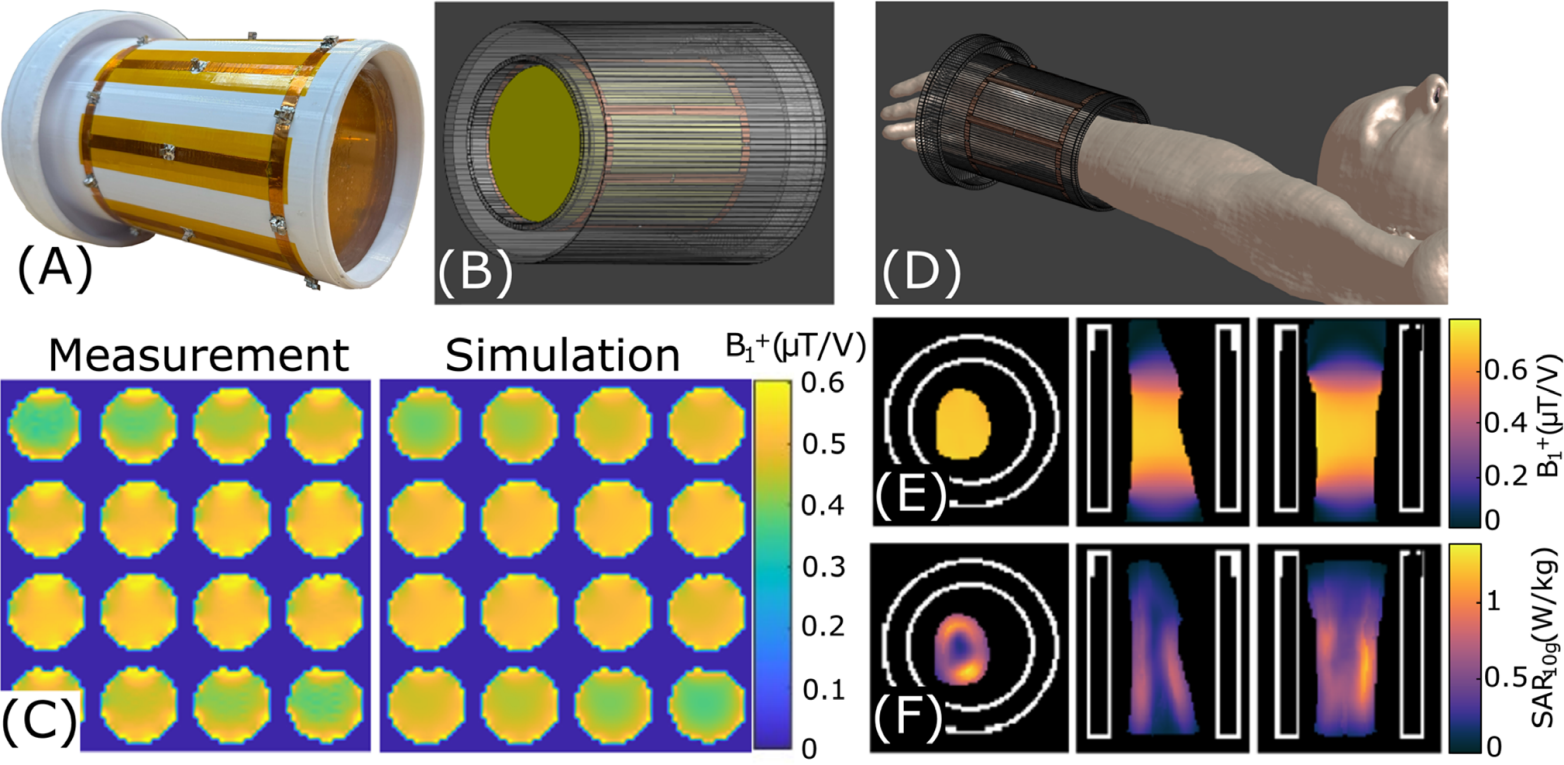
RF coil is shown in (A) as a photo of the coil as constructed and in (B) as a coil model in Sim4Life with (C) comparisons of the B_1_^+^ field as measured (left) and simulated (right) showing good agreement. (D) shows a simulation setup for an in vivo wrist experiment with (E) B_1_^+^, and (F) SAR_10g_ maps simulated in the wrist.

Because of the unique frequency of sodium at 10.5 T, a new RF coil was constructed for this project. The RF coil developed is a quadrature‐driven 8‐rung high‐pass mode bandpass birdcage coil (Figures 2 and 5A). It has a diameter and length of 10 cm. Conductors are constructed of ¼″ (6.35 mm) wide copper tape adhered to a former, which is 3D printed from polycarbonate filament.

At 10.5 T, all RF coils must be added to the 10.5 T investigational device exemption (IDE) through the U.S. FDA, since magnetic field strengths above 8 T are not considered nonsignificant risk. As such, B_1_^+^ maps were acquired of a homogenous cylindrical phantom constructed from acrylic (72 mm L × 88 mm ID) composed of a saline PVP solution (651 g PVP, 17.8 g NaCl, 0.48 g NiCl_2_/1000 g H_2_O) with human tissue mimicking EM properties which were measured with a DAKS‐12 dielectric probe (Schmid & Partner Engineering AG, Zurich, Switzerland) to be: *σ* = 0.499 S/m, *ɛ*_r_ = 56.0.

The coil was simulated using Sim4Life (Zurich MedTech AG, Zurich, Switzerland) using a model of the coil (Figure 5B) and a test phantom used for comparison to the experiment. Simulations were repeated on the Duke model (Figure 5D). Experimental B_1_^+^ maps were acquired using an AFI sequence [53] on the 10.5 T system using the same test phantom. This data was used to validate simulations, and this became the basis for a submission to the FDA to add this coil to our 10.5 T IDE via an amendment.

### 2.8 MRI Experiments

All MRI experiments were performed on a Siemens MAGNETOM 10.5 T system (60 cm open bore magnet manufactured by Agilent Technologies). This system is equipped with Siemens SC72 body gradient (70 mT/m max amplitude, 200 T/m/s max slew rate, 2nd order shims at 20 A/CH). X‐nuclei experiments are run in single‐channel/combined mode with an 8 kW broadband RFPA (Comet LPPA 22080 W, Wünnewil‐Flamatt, Switzerland) with ˜6.5 kW of power available at the patient table.

**FIGURE 6:**
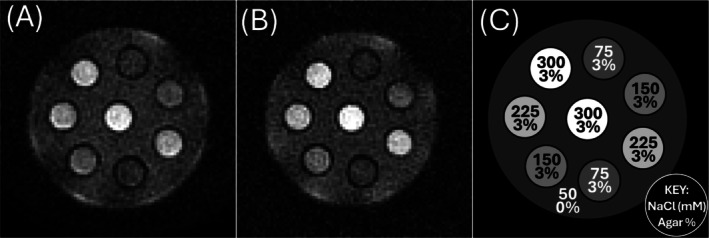
Sodium images of a phantom acquired using (A) SWIFT (HS1 pulse) acquisition with 1.5 mm isotropic resolution, TR: 40 ms, RF on/off: 20 μs/20 μs, projections: 16384, FA: 30 deg., FOV: 192 × 192 × 192 mm^3^, TA: 11 min, and (B) UTE acquisition with 1.5 mm isotropic resolution, TR/TE: 40 ms/150 μs, projections: 16500, FA: 45 deg., FOV: 192 × 192 × 192 mm^3^, TA: 11 min. (C) Phantom solution composition was 50 mM of NaCl with 0% agar in the large container with test tubes containing various concentrations of NaCl, as shown with 3% agar.

Imaging was performed on a phantom with varying concentrations of sodium and agar, details of which are depicted in Figure 6C. A pair of proton dipole elements [54] was used for B_0_ shimming prior to X‐nuclei imaging [26]. After standard localizers, both SWIFT and UTE images were acquired with sequence parameters (SWIFT/UTE) matched as closely as possible: 40 ms TR, 20/150 μs TE, 30/45° flip angle, 1.5 mm isotropic resolution, 192 × 192 × 192 mm^3^ field of view (FOV), 16384/16500 center‐out radial projections, and 11 min scan time. In the SWIFT acquisition, the hyperbolic secant (HS1) pulse used for excitation had 32 gaps (20 μs transmit sub‐pulses interleaved with 20 μs receive gaps) for a total duration of 1.28 ms and bandwidth of 23.75 kHz (= 0.95 × 25 kHz), where the excitation bandwidth was set to 95% of the gapping frequency (25 kHz = 1/40 μs) to avoid crosstalk of the baseband and sidebands in the gapped HS1 excitation profile. For UTE, the RF pulse duration was 100 μs with TE defined from the pulse center to the beginning of the ADC. Here, an additional 100 μs dead time was inserted to allow for coil ring down. The native SWIFT acquisition is acquired with a readout bandwidth of 1 MHz (1 μs dwell time per sample point), where data was sampled with gradient amplitudes of 11.6 mT/m during the gapped HS1 pulse and 5.9 mT/m after the RF pulse, resulting in readout bandwidths of 25 kHz and 12.8 kHz during and after the RF pulse, respectively. The gradient amplitude transitioned during 50 μs with a slew rate of 114 mT/m/ms, which resulted in a total readout time of ˜5 ms. The oversampled SWIFT data with the two gradient amplitudes was transformed to the frequency domain with nonuniform FFT for each radial view and then down‐sampled during image reconstruction [55]. Sample point dwell time for the UTE sequence was 78 μs for 64 ADC samples with a readout duration of ˜5 ms using a trapezoidal gradient with a slew rate of 180 mT/m/ms and amplitude of 5.9 mT/m, resulting in an acquisition bandwidth of 12.7 kHz. Data reconstruction for UTE was performed offline using MATLAB (R2025a, The Mathworks Inc., Natick, Massachusetts, USA) and the Berkeley Advanced Reconstruction Toolbox (BART) to perform density correction and nonuniform FFT [56].

In vivo imaging was performed on the left wrist of a single male subject with FDA and IRB approval to demonstrate the utility of sodium SWIFT for MSK imaging. The SWIFT sequence parameters were identical to those in the phantom scan except for: 20 ms TR, 37.5° flip angle, and 5.5 min scan time.

## 3 Results

### 3.1 Switching Speeds

The high‐speed optical trigger performance demonstrated by the oscilloscope traces of the input (OSC0) and output (Trigger), along with the gapped RF pulse and the RFPA unblank, is shown in Figure 1A.

**FIGURE 7:**
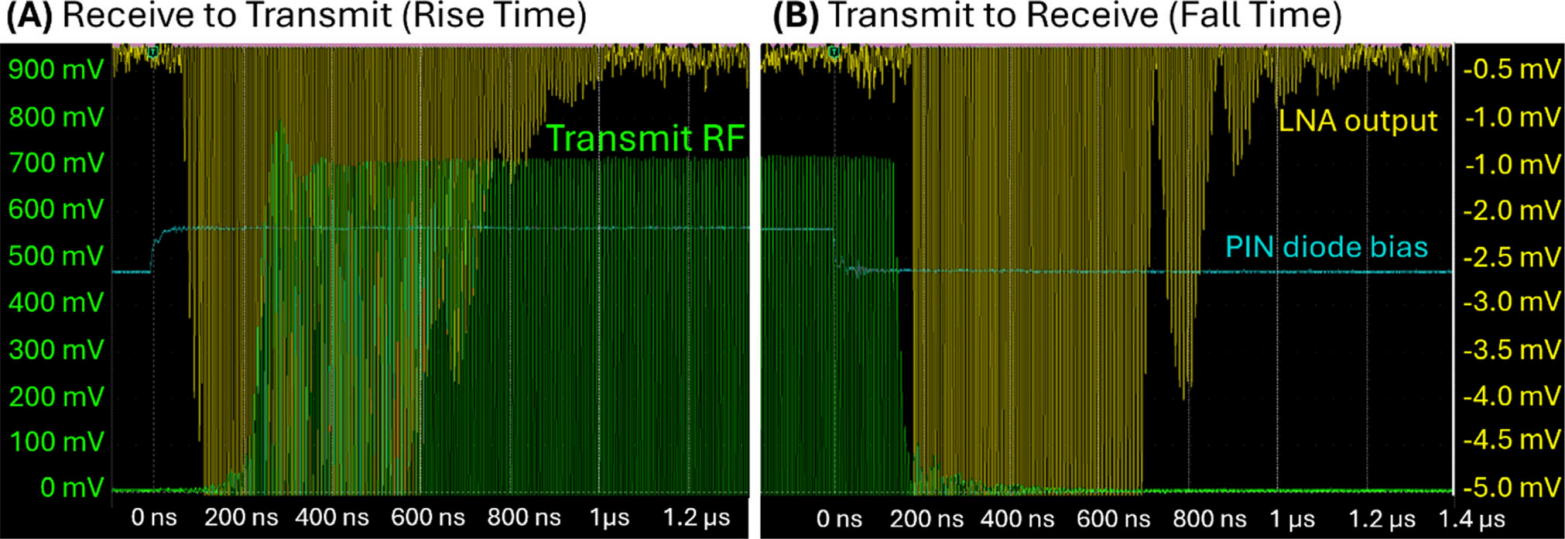
Switching speeds measured of the entire system with receive to transmit switching (rise time) shown in (A) and transmit to receive switching (fall time) shown in (B). The PIN diode bias voltage supplied to the PIN diodes is shown by the cyan trace. PIN diode switching speed measurements are made with an RF signal injected at the input of the switchable attenuator and measured at the coil port (green trace). Practical switching speed from transmit to receive is determined by the time it takes for spurious emissions from the LNA output to drop below the instrument noise floor (yellow trace).

Rise (receive to transmit) and fall (transmit to receive) times, as defined by the start of the TTL edge to 90% or 10% of max RF amplitude, respectively, were measured at < 0.6 μs (see the green trace of Figure 7). Fall time proved the most critical performance specification. The output from the LNA must be free from transients (below the instrument noise floor) as quickly as possible. In practice, this was much slower than the time it took to observe a drop in the envelope of an RF waveform injected through the T/R switch, which was the measurement setup used for switching time tests (as described in Supporting Information). For the complete RF switch (switchable attenuator + T/R switch), the practical fall time was measured at 1.7 μs (yellow trace of Figure 7). At the acquisition bandwidth of 1 MHz, this would require us to discard two sample points at the beginning of each receive gap during image reconstruction. In practice, the images presented below were reconstructed after discarding four sample points from the beginning and end of each receive gap, leaving 12/20 sample points for reconstruction.

### 3.2 T/R Isolation

T/R isolation achieved independently by both the switchable attenuator and the T/R switch was measured to be 60 dB in receive mode. In transmit mode, > 55 dB of isolation is achieved to the LNA, which will protect it beyond the maximum power available from the RFPA. Insertion loss in transmit mode of the attenuator is 0.49 dB, and on the T/R switch is 0.22 dB for a total of ˜0.7 dB.

**FIGURE 8:**
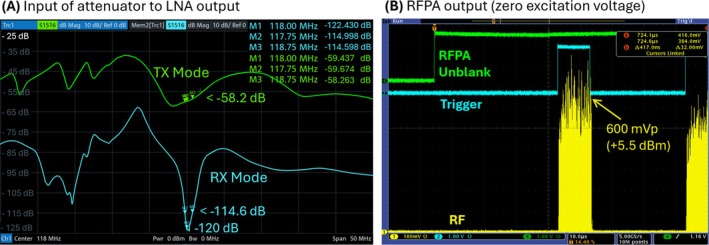
(A) S_21_ measurement on a VNA from the input of the switchable attenuator to the output of the LNA with the RF coil connected to the coil port of the T/R switch. The preamp gain of 25 dB is calibrated out of this measurement. (B) Oscilloscope traces showing the RFPA unblank signal in green, high‐speed trigger output in cyan, and RF measured at the output of the coil port of the T/R switch in yellow. When unblank goes high, there is no measurable increase in noise, showcasing the high T/R isolation of the hardware. When the trigger goes high for the transmit sub‐pulse, the RFPA noise is seen passing to the coil port. This measurement is made with zero excitation voltage to demonstrate the high level of noise on the unblanked RFPA.

**FIGURE 9:**
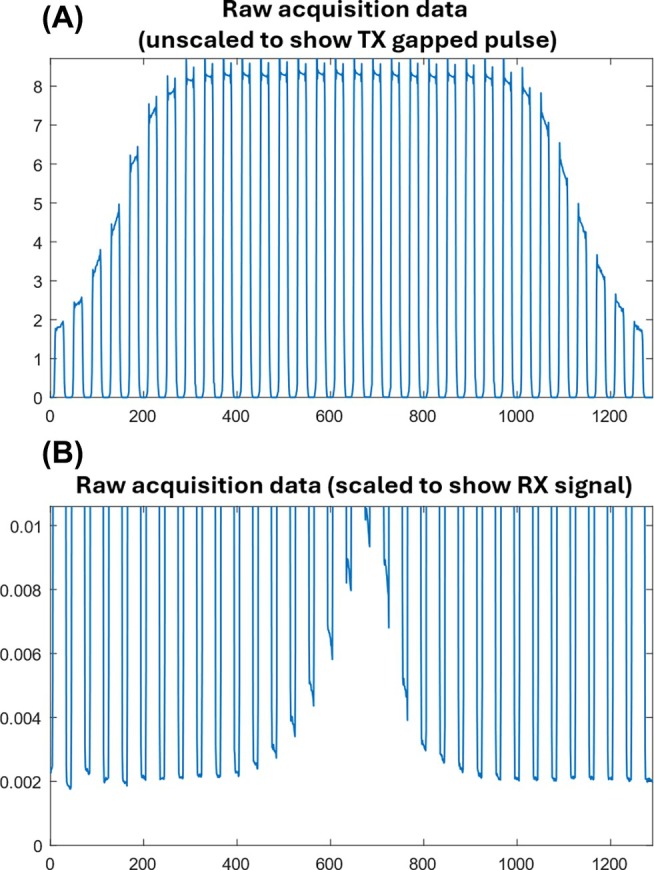
Raw data acquired from a SWIFT acquisition. The envelope of the gapped HS1 pulse is visible in the unscaled data (A), and the NMR signal is visible in the receive gaps in the scaled data in (B). Note that the envelope shown in (A) is flattened because it has undergone signal compression due to the excessive RF input to the LNA during the transmit sub‐pulses.

Due to the inability to blank the RFPA, T/R isolation during the receive gap was a critical performance metric. The amplitude of the unblanked RFPA noise is 600 mVp, or +5.5 dBm. Isolation in receive mode across the 1 MHz acquisition bandwidth was measured to be > 115 dB, as shown in Figure 8A. The resulting noise level arriving at the LNA from the RFPA is lower than −110 dBm. The effectiveness of the high T/R isolation on the RFPA noise is demonstrated with a 0 V RFPA pulse. The full amplitude of the RFPA noise is seen in the transmit sub‐pulses, but nothing is observed during the receive gaps (Figure 8B). To confirm that RFPA noise was sufficiently attenuated, it proved useful to inspect raw data acquired from the receiver. While the transmit sub‐pulses at amplitudes of the HS1 pulse envelope (which itself is flattened due to LNA compression) are visible in the data that is scaled to the maximum value (Figure 9A), the NMR signal without any excessive noise is clearly visible in receive gaps when the y‐axis of the data is scaled down (Figure 9B).

### 3.3 RF Coil Validation

B_1_^+^ maps acquired experimentally agreed well with B_1_^+^ fields calculated from EM simulations of the birdcage coil. These results are summarized in Figure 5C. B_1_^+^ and SAR10g field maps are shown for a human wrist in Figure 5E,F.

### 3.4 Imaging

Representative axial UTE and SWIFT images of a test phantom with varying sodium concentrations are shown in Figure 6.

**FIGURE 10:**
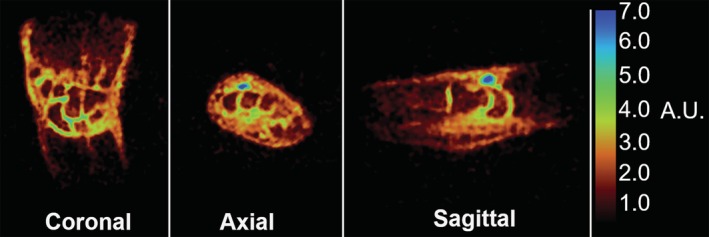
Pilot in vivo images of the left wrist of a human volunteer with a reconstructed resolution of 1.5 mm isotropic. Articular cartilage of the radiocarpal and intercarpal joints is clearly shown by high‐intensity signal outlining the carpal bones due to the naturally occurring high sodium concentrations in this tissue. Carpal bones demonstrate expected voids of signal. An incidental finding of a ganglion cyst can be identified as the approximately 5 mm diameter hyperintensity in blue at the dorsal aspect of the wrist. These are signal intensity images with arbitrary units, scaled as shown by the color bar on the right.

To showcase in vivo MSK application of sodium SWIFT for the evaluation of articular cartilage, an axial, sagittal, and coronal image of a human wrist is presented in Figure 10.

## 4 Discussion

The two most demanding specifications for the hardware developed herein were fast switching speeds and high isolation. All the usual limitations of MRI hardware for clinical imaging existed. Designs must be non‐magnetic or contain low magnetic material. For example, some components contained a nickel underplating. Because of the distance of the RF switch from the RF coil, *B*_0_ inhomogeneities were not a concern. Components must handle high RF power on transmit.

### 4.1 Switching Speed

Sub‐microsecond switching speeds were initially achieved through careful selection of the PIN diode. While MACOM is not the only manufacturer of MRI‐compatible PIN diodes, the diodes considered for this design were those that had a proven track record in previous designs familiar to the authors. It proved necessary to choose a PIN diode with a short carrier lifetime and a thin intrinsic region width. The trade‐offs being a propensity of a PIN diode to be switched by RF and lower reverse voltage breakdown. The latter issue was not a concern in this design. After initial attempts to use shunt diodes that were reverse‐biased during transmit, all circuitry was modified so that PIN diodes were forward‐biased during transmit. Component failures suffered with this circuit topology were primarily due to initial synchronization problems between the RF pulse and trigger signal.

The RF hardware developed for SWIFT relies upon the MACOM MA4P504‐1072T PIN diode for switching purposes. While this part is not intended for MR applications that are in‐bore, having nickel terminations, which are magnetic, it has other favorable specifications when compared to other common PIN diodes used in MRI. Rise and fall times of this diode and other common PIN diodes were measured prior to T/R switch development, and this diode compared favorably. The intrinsic region width and minority carrier lifetime of the MA4P504 allow for sub‐microsecond switching, while still being slow enough to act as an RF switch. Secondary considerations were a small off‐state capacitance necessary to produce high isolation when reverse biased (receive mode), as well as a low series resistance to minimize insertion loss when forward biased (transmit mode).

Diodes are driven in parallel to increase switching speed. This increases the necessary drive current, since each diode is supplied with its own 100 mA, but reduces the necessary drive voltage. When driven in series, the switching speeds are markedly slower [51].

A secondary issue affecting switching speeds appeared to be ringing. Up to four microseconds during receive appeared to be corrupted by switching transients. These transients were diagnosed as resulting from inductive ringing in the PIN diode DC path. A reduction of the path inductance resulted in lower inductive ringing. Previously, relatively large inductance (3900 nH) ceramic core RF chokes were used in the RF circuitry. The benefit of using these RF chokes was high isolation at the frequency of interest, with broadband isolation over most RF frequencies. The significant drawback was high path inductance. These RF chokes were substituted for LC resonant tanks for RF choking. A parallel combination of a 12 pF capacitor and a 150 nH inductor resonated at 118 MHz to produce a high impedance while offering a much lower path inductance. Subsequently, transients were shorter during switching, allowing for a clean output from the LNA after 1.7 μs. The significant drawback to LC choking is that it is narrowband by nature, and each choke requires fine‐tuning to the correct frequency. Because both the inductor and capacitor chosen have a relatively low reactance at 118 MHz, an improperly adjusted choke has a significant impact on the RF performance of the circuits. Another unfortunate side effect was the unintentional conversion of the switchable attenuator into a bandpass filter. Reverse‐biased PIN diodes present a very small coupling capacitance between what effectively become several LC stages of a direct‐coupled series capacitor bandpass filter, which is poorly tuned to 118 MHz. The result was the switchable attenuator readily passing this frequency while rejecting all others, which was the opposite of what was intended. Several LC tanks were implemented across the PIN diodes to spoil this coupling behavior of the PIN diodes.

### 4.2 T/R Isolation

High T/R isolation was initially achieved through the use of numerous PIN diodes in series along the transmit path. Because each presented a small series capacitance of 0.5–1 pF when reverse‐biased, this reactance was resonated out to a high impedance through the addition of more capacitance in parallel and an inductor. Extremely high isolations could be achieved in this way. The limitation became RF bypassing these PIN diodes entirely via the DC lines used to switch the diodes. While a conventional approach to preventing this might be several series RF chokes with several filtering or bypass capacitors, this presented problems from a switching speed perspective and was not an option available, which would meet the specifications for this RF switch. While LC chokes provided high isolation, neighboring LC chokes coupled to one another. Consequently, LC chokes were shielded from one another, whenever possible, with copper foil, which was referenced to the RF ground.

Another major barrier to high T/R isolation was the RF coil itself. The coil, despite being a shielded birdcage, proved an effective enough radiator or antenna. Once connected to the RF switch, it defeated much of the achieved isolation. This necessitated housing all RF hardware inside RF shielded enclosures. Despite this effort, it was found that RF would couple onto the DC wiring, which ran from the PIN diode driver to the switchable attenuator and T/R switch. RF, which coupled onto this cable, would significantly cut into isolation. The solution we arrived at was the implementation of a filter immediately inside the penetration to the shielded box. An RF shielded LC notch filter was constructed. The forward and return DC paths made up conductors one and two of a trifilar wound inductor. The third conductor was resonated with a variable capacitor and tuned to 118 MHz. This effectively notched out any RF within the bandwidth of the RF coil. This device was implemented at the penetration points to both the switchable attenuator and the T/R switch.

### 4.3 Imaging

Phantom imaging confirmed that SWIFT achieved comparable image quality to UTE imaging. Both imaging sequences were sensitive to *B*_0_ inhomogeneity, necessitating *B*_0_ shimming to produce phantom images with well‐resolved details such as the thin plastic walls of the tubes containing agar. What a phantom does not demonstrate, however, is the benefit that SWIFT has on imaging tissues with extremely short relaxation times, such as articular cartilage. As Zbýň et al. estimated previously, $T_{2S}^{*}$ in the pediatric knee can approach 0.4 ms [26]. While the UTE sequence used in this work still has a very short TE, at 150 μs, SWIFT can achieve roughly one order of magnitude shorter TE (20 μs) by sampling the NMR signal during excitation with the gapped frequency‐swept RF pulse, which can preserve 49% more signal from the $T_{2S}^{*}$ spins in the pediatric knee.

In vivo imaging of a human wrist provided excellent results. A 1.5 mm isotropic resolution image acquired in 5 min provided clear detail of the wrist anatomy, including low signal regions where wrist bones are located and a high sodium signal in the articular cartilage. A hyperintensity visible in the image is posited to be an incidental finding of a ganglion cyst. The volunteer disclosed a suspicion of a ganglion cyst, but previous ultrasonic imaging could not confirm the diagnosis.

### 4.4 Limitations

Some limitations emerged throughout the hardware development. As mentioned previously, while the PIN diodes in the switching hardware complete switching in less than 1 μs, the LNA produces spurious emissions up to 1.7 μs after the trigger, which limits the transmit‐to‐receive switching speeds to 1.7 μs due to corruption of the receive signal. This does not limit receive to transmit switching since corruption of the RF sub‐pulse is not a concern. If this were the only limitation, it would amount to the loss of a single additional sample point from each receive gap.

A far greater detriment was not a result of the hardware switching speed, but rather the console itself. Some post‐processing or filtering of the acquired signal resulted in smoothing of the raw data. This smoothing resulted in corruption of four sample points at the beginning and end of each receive gap. We believe the culprit is digital filtering, which is performed after the ADCs on the receiver. It is not possible for this to be a result of our hardware, since there is corruption at the end of each receive gap prior to any trigger signal being sent to our hardware. In practice, this effect causes a substantial loss of 8 sample points every receive gap (only three of which are corrupted by our hardware switching time). Consequently, only 12/20 sample points per receive gap could be used for image reconstruction. If a workaround to this can be found, 17/20 sample points (an information increase of almost 50%) could be used per receive gap.

As for hardware constraints, while the long‐term average RF power used is below what the PIN diode can dissipate, the RF pulse is long enough to cause excessive heating at the semiconductor junction. This is evident as diodes would start to see a degradation in their reverse bias resistance. A barely detectable DC current would leak through, and RF would start to bypass the diodes once they were damaged. As such, maximum voltage was limited to 300 V. The consequence of this was that imaging samples much larger than an extremity, such as the wrist, became difficult or impossible with this setup. This is a relatively straightforward problem to overcome in future work and simply calls for several parallel diodes so power dissipation is divided between numerous components instead of one.

With respect to optimizing and comparing UTE and SWIFT, there were limitations in imaging parameters due to peak power and SAR constraints, given the remaining hardware limitations. In terms of SAR constraints, due to the sample rate of the RF pulse envelope by the console, RF power delivered to the coil is overestimated by up to ˜50%. Theoretically, however, for the same bandwidth and flip angle, an ungapped SWIFT HS1 pulse and a UTE square pulse will have the same relative energy and SAR for matching TR. However, when gapping the pulse with even periods of RF on/off, the SAR doubles for SWIFT due to increased sub‐pulse amplitudes to maintain the target flip angle [57]. Additionally, the energy increases proportionally to the bandwidth (i.e., 25 kHz/10 kHz), which is another 2.5‐fold increase in SAR. Therefore, for the sequences comparing SWIFT and UTE in the phantom, there was a 5‐fold increase in SAR for SWIFT. To reduce SAR during the SWIFT excitation, the frequency sweep of the frequency‐modulated pulse could be reduced. This highlights one advantage of SWIFT, where the bandwidth is not coupled with the pulse duration [58]. Another option is to increase the RF duty cycle, which reduces sub‐pulse amplitudes and total pulse energy. An RF on/off of 80/20 μs, for example, would yield a similar power deposition as UTE while still maintaining a shorter echo time than UTE. Currently, our hardware does not allow switching of RF on periods longer than 20 μs, which limited the exploration of these strategies in the current study.

Another consideration when imaging low‐γ nuclei is the limitations in peak available voltages, either due to RF coil component tolerances or limited available power from the RF amplifiers. In the phantom examples shown in Figure 6, the bandwidth of the UTE excitation pulse could not be made short enough (i.e., 40 μs) to match the 25 kHz bandwidth of the HS1 SWIFT excitation without reducing the flip angle to 28°, thus greatly reducing SNR. For the frequency‐swept pulses in SWIFT, the option exists to stretch the HS1 pulses by previously defined non‐linear arguments to the modulation functions [58]. These HS*n* pulses reduce the peak power by flattening the amplitude modulation function and adjusting the frequency sweep. It is expected that an HS2, HS4, and HS8 excitation pulse would reduce the required excitation voltage by 34.7%, 48.0% and 53.9%, respectively, compared to the HS1, while roughly maintaining SAR.

Continued exploration of SWIFT and its variants for sodium imaging is required to understand its tradeoffs compared to UTE and other widely used 3D sodium acquisition strategies, such as twisted projection imaging (TPI) [59] and density‐adapted radial projection (DARP/DA‐3DPR) [27]. Given the sub‐microsecond switching presented in this work, these evaluations will be the focus of future investigations, along with exploring setups for investigating proton (^1^H) imaging and potentially other nuclei.

## 5 Conclusion

In this work, we developed RF hardware that simultaneously achieves fast switching while preserving very high transmit‐to‐receive isolation. The hardware enables rapid switching between transmit and receive, which in turn allows the utilization of zero echo time imaging techniques such as SWIFT. The capability of this hardware and the utility of SWIFT for sodium imaging of MSK structures were showcased by imaging the left wrist of a human volunteer. Future work includes translating this work to proton imaging at 3 T, 7 T, and 10.5 T. Other x‐nuclei might also benefit from zero echo time imaging techniques and solutions presented in this technical development.

## Funding

This work was supported by the National Institutes of Health, P41 EB027061, S10 RR029672, R01 EB034746, and R01 EB029985.

## Conflicts of Interest

Dr. Moeller is entitled to royalties through the University of Minnesota for products related to the research described in this paper. These relationships have been reviewed and managed by the University in accordance with its conflicts of interest policies.

## Supporting information


**Figure S1:** T/R switch test circuit with the PIN diode bias supplied (a) in series and (b) in parallel.
**Table S1:** measured rise and fall times of four PIN diodes from MACOM are shown in the first two rows. These times are measured from the trigger edge to 90% of RF max amplitude (rise time) or 10% of RF max amplitude (fall time). The second two rows are carrier lifetime and intrinsic region width provided by manufacturer datasheets. *not provided on datasheet. **Datasheet lists 2 mm, assumed to be 2 mil.

## Data Availability

The data that support the findings of this study are available from the corresponding author upon reasonable request.
