# Supplementary material for: A Sub‐Microsecond Switch Enabling SWIFT 23Na Imaging at 10.5 T
Source: Magn Reson Med. 2026 Jun 8;96(4):1991–2003. doi: 10.1002/mrm.70461 (PMC13419349; doi:10.1002/mrm.70461)
Supplement: Supplementary file 1 — Figure S1: T/R switch test circuit with the PIN diode bias supplied (a) in series and (b) in parallel. Table S1: measured rise and fall times of four PIN diodes from MACOM are shown in the first two rows. These times are measured from the trigger edge to 90% of RF max amplitude (rise time) or 10% of RF max amplitude (fall time). The second two rows are carrier lifetime and intrinsic region width provided by manufacturer datasheets. *not provided on datasheet. **Datasheet lists 2 mm, assumed to be 2 mil. [file MRM-96-1991-s001.docx]

## **Supporting Information**

A sub-microsecond switch for ^23^Na imaging at 10.5T

## PIN Diode Testing

### Methods

Several commonly used MRI PIN diodes in our lab were previously tested and compared^1^ in terms of rise and fall time and were the basis by which we selected the MA4P504-1072T PIN diode for development of the RF switching hardware in this work. That data is reproduced here for the reader’s convenience.

The diodes compared were the MA4P7470F, MA4P7104F, MA4P1250NM, and MA4P504 all in the 1072T package. These tests were performed with T/R switch test circuits each of which had three PIN diodes for RF switching. A signal generator (SMY 02, Rohde & Schwarz, Germany) injected the appropriate RF signal at the TX port with an oscilloscope monitoring the COIL port. Rise time was defined as the time from the TTL trigger edge to 90% of full RF amplitude while fall time was defined as the time from TTL trigger edge to 10% of full RF amplitude. These tests were performed with all diodes driven in series from a shared DC path (Figure S1a) and with each diode driven in parallel and receiving its own DC bias from a common voltage rail (Figure S1b).

### Results

Comparisons of three PIN diodes driven with the DC drive signal wired in series vs parallel showed that the parallel drive yielded 15% faster rise times and 35% faster fall times. Furthermore, testing of several diodes with diverse intrinsic region widths and carrier lifetimes resulted in the measured rise and fall times documented in Table S1. As a result, the MA4P504 diode from MACOM was selected as the basis for further design as it was the only device with a fall time faster than 1 us.


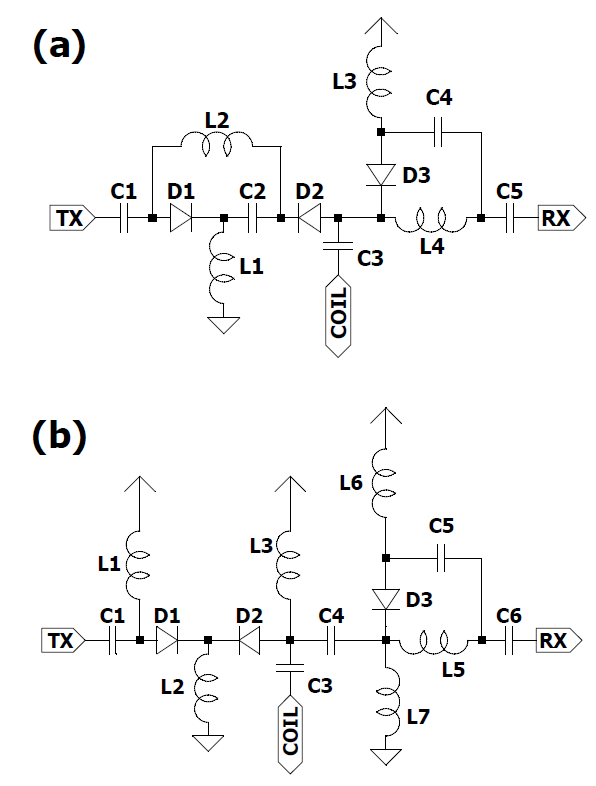


***Figure S1:*** *T/R switch test circuit with the PIN diode bias supplied****(a)****in series and****(b)****in parallel.*

##
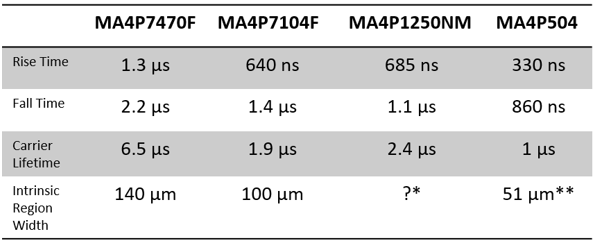


***Table S1:*** *measured rise and fall times of four PIN diodes from MACOM are shown in the first two rows. These times are measured from the trigger edge to 90% of RF max amplitude (rise time) or 10% of RF max amplitude (fall time). The second two rows are carrier lifetime and intrinsic region width provided by manufacturer datasheets. *not provided on datasheet. **datasheet lists 2 mm, assumed to be 2 mil*

## Supporting Information Bibliography:

1. Lagore RL, Auerbach E, Kobayashi N *et al.* Fast transmit/receive switch for SWIFT imaging at 7T*.* *Annual Meeting of the International Society of Magnetic Resonance in Medicine*. (2019).
